# NLRs: Sentinels of innate immunity or cancer culprits?

**DOI:** 10.18632/oncoscience.44

**Published:** 2014-05-26

**Authors:** Ricardo G. Correa, Carl F. Ware, John C. Reed

**Affiliations:** Sanford-Burnham Medical Research Institute, La Jolla, CA

The intersections between innate immunity and cancer have been investigated for decades, with widespread acceptance that chronic inflammation contributes to the pathogenesis or progression of several types of malignancies. Numerous mediators of innate immune responses have been connected to tumor risk through genetic, molecular, and cellular analysis [1]. The NACHT and Leucine Rich Repeat domain containing proteins (NLRs) represent a family of innate immunity proteins responsible for pathogen sensing in the cytosol. Analogous to membrane-associated Toll-like receptors (TLRs), there is evidence that NLRs participate in recognition of both pathogen-associated molecular patterns (PAMPs) and endogenous byproducts of tissue injury (danger associated molecular patterns, DAMPs) [1, 2]. NLRs activate inflammatory signaling pathways including NFκB and MAPK, stress kinases, interferon responses factors (IRFs), autophagy, and caspase-dependent cytokine secretion. A growing body of evidence has associated NLR polymorphisms with several human cancers. However, the precise role of these NLRs in cancer progression is not well defined.

To date, a total of 22 NLRs have been described in humans, but this number rises when considering atypical members that possess alternate domain structures. Currently, a basic characterization of all NLR members is lacking, thus our insights into the diversity of activators of these proteins and the downstream signaling pathways they control are limited to a few of the family members. The most studied NLRs include NOD1 (NLRC1) and NOD2 (NLRC2), which are responsible by the activation of innate immunity signaling pathways through recognition of particular bacterial peptidoglycan derivatives. NOD activation is driven by NACHT domain-mediated self-oligomerization and by CARD-CARD interactions with downstream effectors, including RIP2, which recruits TAB/TAK complexes to activate IκB kinase complex (IKK) and MAPK kinases, resulting in NF-κB and AP-1 signaling activation, respectively [2]. NOD1 and NOD2 have both been defined as seminal players in gut homeostasis, providing protection against colitis and cancer. Indeed, *Nod1*^−/−^ mice show remarkable predisposition for tumor formation using a colitis-associated colon cancer model [3]. Also, breast cancer cells (MCF-7) devoid of *NOD1* expression give rise to larger tumors after injection in immunocompromised (SCID) mice [4]. More recently, *NOD2* gene polymorphisms have been associated with higher predisposition to a number of cancer types, including increased risk of gastric cancer in *H. pylori*-infected patients [5]. These observations have prompted several investigators to consider NODs as possible therapeutic targets for pro-inflammatory diseases and gastrointestinal cancers [2].

**Figure 1:**
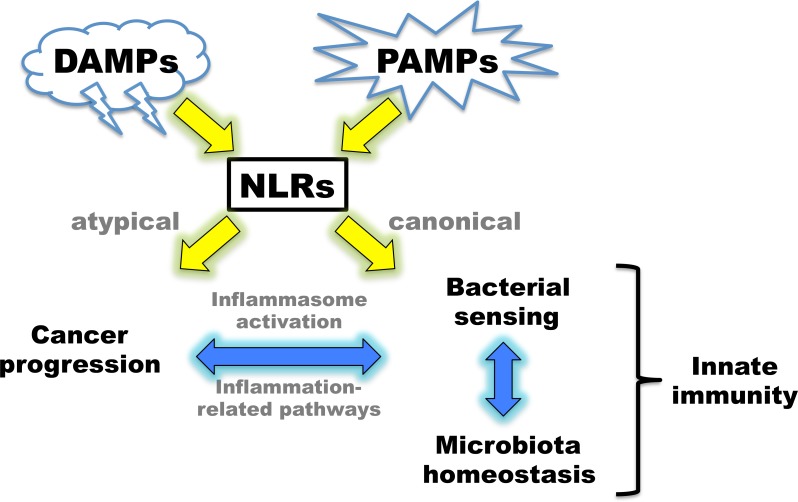
Overview of the connections of NLR family members to innate immunity and cancer progression Potential effects due to functions of atypical and canonical NLRs are indicated.

Another subset of NLR members, including NLRP1, NLRP3 and NLRC4, is responsible by the formation of multimolecular structures dubbed “inflammasomes” that, in combination with adapter protein ASC and protease caspase-1, drive the maturation and secretion of cytokines IL-1β and IL-18. These cytokines contribute to innate immune responses against microorganisms, and they are also important for tissue repair and tumor surveillance at the intestinal mucosal surface [1]. In this regard, it is hypothesized that changes in the composition of the gut microflora function as a prime driver of intestinal tumorigenesis [1]. Consistently, mice lacking the inflammasome-forming NLRs NLRP3 and NLRP6 show increased susceptibility to colorectal carcinoma, possibly due to absence of protective IL-18, as well as because of altered composition and function of microbial species in the gut (dysbiosis) [1].

NLRs have also been implicated in neoplasia beyond the gastrointestinal tract. For example, genetic variants of *NLRP7* are involved in post-molar choriocarcinoma (as indicated in [6]), Also, germline mutation of *NLRP2* is associated with a non-specific organ overgrowth disorder, Beckwith-Wiedemann syndrome (as indicated in [6]). Interestingly, a broad range of somatic mutations affecting many pyrin domain-containing NLRs (NLRPs) have been described in patients with head and neck squamous cell carcinoma (HSNCC) and also associated with increased cancer genome instability [6]. Since a significant portion of HNSCC is comprised of cancer of the oral cavity, changes in the composition and function of the oral microbiota (possibly due to a selective pressure imposed by alterations on NLRP sensing functions) could conceivably contribute to the development of such diseases.

The participation of atypical NLR members in cancer progression has also been extensively studied. The pro-apoptotic protein Apaf-1 is a distant cousin of NLRs, containing a nucleotide-binding oligomerization domain resembling NACHT and also a CARD. Apaf-1, the core component of the apoptosome complex, is mutated in human melanomas, and its depletion contributes to malignant transformation in a mouse model of cancer [7]. Interestingly, at least one NLR family member (NLRP1) has been reported to interact with Apaf-1. More recently, the NLR-like protein NWD1 has been characterized in the context of prostate cancer, where it has been shown to participate in androgen receptor (AR) signaling by mediating AR protein stability as a putative co-chaperone molecule [8]. While possessing the canonical NACHT domain, NWD1 lacks the LRRs required for official membership in the NLR family, possessing instead WD40 repeats akin to those found in Apaf-1.

The conservation of nucleotide-binding NACHT domains within NLR family (including for some atypical members), which typically displays ATPase activity to promote protein self-oligomerization, could be envisioned to constitute a “druggable” site for blocking NLR activities in a variety of maladies. On balance, however, it remains to be determined how detrimental or beneficial various NLRs might be during cancer progression and thus detailed analysis will be required to better delineate the roles of these proteins as sentinels of innate immunity, cancer culprits, or both.
